# Crystal Structure of the FLT3 Kinase Domain Bound to the Inhibitor Quizartinib (AC220)

**DOI:** 10.1371/journal.pone.0121177

**Published:** 2015-04-02

**Authors:** Julie A. Zorn, Qi Wang, Eric Fujimura, Tiago Barros, John Kuriyan

**Affiliations:** 1 Department of Molecular and Cell Biology, University of California, Berkeley, California, United States of America; 2 California Institute for Quantitative Biosciences, University of California, Berkeley, California, United States of America; 3 Howard Hughes Medical Institute, University of California, Berkeley, California, United States of America; 4 Department of Chemistry, University of California, Berkeley, California, United States of America; 5 Physical Biosciences Division, Lawrence Berkeley National Laboratory, Berkeley, California, United States of America; Yale University School of Medicine, UNITED STATES

## Abstract

More than 30% of acute myeloid leukemia (AML) patients possess activating mutations in the receptor tyrosine kinase FMS-like tyrosine kinase 3 or FLT3. A small-molecule inhibitor of FLT3 (known as quizartinib or AC220) that is currently in clinical trials appears promising for the treatment of AML. Here, we report the co-crystal structure of the kinase domain of FLT3 in complex with quizartinib. FLT3 with quizartinib bound adopts an “Abl-like” inactive conformation with the activation loop stabilized in the “DFG-out” orientation and folded back onto the kinase domain. This conformation is similar to that observed for the uncomplexed intracellular domain of FLT3 as well as for related receptor tyrosine kinases, except for a localized induced fit in the activation loop. The co-crystal structure reveals the interactions between quizartinib and the active site of FLT3 that are key for achieving its high potency against both wild-type FLT3 as well as a FLT3 variant observed in many AML patients. This co-complex further provides a structural rationale for quizartinib-resistance mutations.

## Introduction

FMS-like tyrosine kinase 3 (FLT3) is a receptor tyrosine kinase that is expressed in both normal and malignant lympho-hematopoietic cells and is important for immune response and stem cell proliferation [1]. It belongs to a family of receptor tyrosine kinases that includes platelet-derived growth factor receptors α and β (PDGFR-α and PDGFR-β), colony-stimulating factor 1 (or FMS) receptor, and the stem cell factor (SCF) receptor c-Kit [2]. The characteristic domain organization of these receptors includes an extracellular module, a transmembrane (TM) helix, and an intracellular module that consists of a juxtamembrane (JM) segment, a kinase domain with a kinase insert region, and a C-terminal tail. Previous structural studies on the intracellular domains of c-Kit, FLT3, and the related vascular endothelial growth factor receptor (VEGFR) and Eph receptor have revealed the importance of the juxtamembrane segment for stabilizing the kinase in an inactive, autoinhibited state [3–7].

The shift from an inactive to an active conformation of the kinase domain is stimulated upon ligand binding to the extracellular module to promote receptor dimerization [8,9]. This brings the intracellular modules into close proximity to allow the kinase domain to catalyze the transfer of a phosphate group from adenosine triphosphate (ATP) to tyrosine residues in the juxtamembrane segment of FLT3 [3]. This releases the autoinhibitory interactions and stabilizes the active kinase, which subsequently autophosphorylates additional tyrosine residues within the intracellular module of FLT3, including Tyr 842 in the activation loop to help stabilize an active conformation [3,6]. Phosphorylation of tyrosine residues in the C-terminal tail and the kinase insert region serve as recruitment sites for downstream substrates to initiate signaling pathways.

The deregulated activation of FLT3 due to mutation or overexpression is linked to the progression of acute myeloid leukemia (AML) and is associated with poor prognosis [10,11]. The internal tandem duplication (ITD) mutations within the juxtamembrane segment contribute to the majority of FLT3 activating mutations in AML. While this insertion can vary in length, the ITD mutations generally result in activation of FLT3 due to release of autoinhibition from the juxtamembrane segment. Additional point mutations in FLT3, which are thought to stabilize the active conformation, have also been identified in AML patients. The most prevalent of these mutations occur at Asp 835 in the activation loop. Typically, cancer cells with activated FLT3 variants become reliant on FLT3 for growth, and therefore, are susceptible to FLT3-targeted inhibitors [10,12].

For the past 20 years, drug discovery efforts have pursued the development of kinase inhibitors to block the aberrant activation of kinases associated with the cancer progression, as observed for FLT3 in AML [13,14]. Over 20 small molecules are now clinically approved and more than 150 additional kinase inhibitors are in clinical trials. In a recent review, we described the interactions that these clinically approved inhibitors exploit in the kinase active site [15]. In particular, FLT3 is potently inhibited by small molecules composed of a diaryl urea core scaffold, which were found to be efficacious in mouse models of the disease [16]. Chemical optimization of these compounds led to the discovery of quizartinib or AC220, which exhibits both selectivity for and potency against FLT3 [17,18]. In fact, quizartinib is currently in clinical trials and has shown promising results as a treatment for AML. However, drug resistance mutations have emerged in response to quizartinib treatment [19].

The lack of a co-crystal structure of quizartinib bound to FLT3 impedes efforts at improving FLT3 inhibitors. We now report the determination of such a structure. We had previously used modeling and molecular docking to predict how quizartinib binds to FLT3, and had found two high-scoring orientations of the drug in the kinase active site, with one preferred by the modeling procedure over the other [19]. The co-crystal structure reported here demonstrates that the drug-binding mode that was less favored in our previous modeling is the correct one. The structure also helps clarify the mechanism of drug resistance mutations, and provides a route forward for further improvement of FLT3 inhibitors.

## Materials and Methods

### Cloning and Expression

A vector containing the full intracellular domain of FLT3 was a gift from the Neil Shah lab at the University of California, San Francisco (UCSF). The intracellular domain of FLT3 (residues 564–994) was cloned into the pFastbacHTa (Invitrogen) insect cell expression vector, which contains an N-terminal His_6_-tag with a TEV protease cleavage site. A portion of the kinase insert (residues 711–761) was removed from this intracellular domain construct. The recombinant virus was generated from FLT3 (564–994, Δ711–761) and amplified to a high titer viral stock. Sf21 cells were grown to 2 x 10^6^ cells/ mL and infected with virus for 48–72 hours at 27°C. Cells were harvested and stored at -80°C until thawed for purification.

### Protein Purification

Thawed cell pellet was resuspended in lysis buffer (50 mM Tris, pH 8.0; 5% glycerol) with protease inhibitors. The cells were lysed using a cell press, and clarified upon centrifugation at 40,000 rpm for 90 minutes. The supernatant was filtered and loaded onto a Ni-NTA affinity column (GE Healthcare Life Sciences). The column was washed with buffer A (20 mM Tris, pH 8; 500 mM NaCl; 5% glycerol), and eluted over a gradient of buffer B (20 mM Tris, pH 8; 500 mM NaCl; 5% glycerol; 250 mM imidazole). Fractions containing FLT3 were pooled; β-mercaptoethanol (Sigma-Aldrich) was added to a final concentration of 1 mM; and TEV protease was incubated with protein overnight at 4°C. The sample was buffer exchanged into buffer A, and a subtractive Ni-NTA column was run to remove impurities and TEV protease. The flow-through was collected, concentrated, and further purified on a Superdex 200 16/60 column (GE Healthcare Life Sciences) in 20 mM Tris, pH 8; 150 mM NaCl; 5% glycerol. Fractions containing FLT3 were pooled, dithiothreitol (Gold Biotechnology) was added to a final concentration of 1 mM, and the sample was concentrated. Concentrated protein was aliquoted, frozen, and stored at -80°C.

### Crystallization and Structure Determination

The FLT3 protein (10 mg/ml, in buffer containing 20 mM Tris-HCl pH 8.0, 150 mM NaCl, 1 mM DTT and 5% glycerol) was mixed with 0.25 mM quizartinib (LC Laboratories) and incubated for 1 hour at room temperature. Co-crystals of FLT3-quizartinib were grown using sitting-drop vapor diffusion. 0.2 μl protein stock solution was mixed with 0.2 μl reservoir solution (0.1 M Tris-HCl pH 7.5, 32% Polyethylene glycol monomethyl ether 3360) using the Crystal Phoenix Liquid Handing system, and the drop was equilibrated at 20°C. The crystals were cryoprotected with the addition of 20% glycerol (v/v) and X-ray diffraction data were measured at the Lawrence Berkeley National Laboratory Advanced Light Source, Beamline 8.2.2. Data were integrated and scaled using HKL2000 [20]. The dataset was anisotropic, diffracting to 2.6 Å in the a and c direction and 3.4 Å in the b direction. We performed anisotropic scaling in Phenix to partially correct for the diffraction anisotropy. A molecular replacement solution was found with Phenix using two C lobes of the autoinhibited FLT3 (1RJB) as an initial search model followed by two N lobes of the autoinhibited FLT3 (1RJB) lacking the juxtamembrane segments [21]. Structure building and refinement was performed with Coot and Phenix [21,22]. The final model has been refined to an R-value of 29.7% (free R-value of 32.0%) at 3.2 Å resolution (see Table 1). The coordinates are deposited in the protein data bank (PDB 4XUF).

**Table 1:** Structure determination and refinement of FLT3 bound to quizartinib.

**Data collection**	
	Native
Wavelength (Å)	1.0000
Space group	P2_1_2_1_2_1_
Cell dimensions	
* a*,*b*,*c* (Å)	48.73, 75.49, 153.97
Resolution (Å)	50.0–3.2*
*R*_sym_ (%)	20.9 (59.7)
*I*/σ(*I*)	4.7 (2.0)
*CC*_1/2_	97.5 (69.8)
Completeness (%)	94.3 (77.1)
Redundancy	3.5 (2.9)
Wilson B factor	75.04
**Refinement**	
Resolution	46.46–3.2
Reflections used	9310
*R*_free_ reflections	932
*R*_work_/*R*_free_	0.297/ 0.320
No. Atoms	
* *Protein	4333
* *Ligands	80
* *Water	2
Average *B*factors	
* *Protein	68.2
Root mean square deviation from ideality	
* *Bonds (Å)	0.004
* *Angles (°)	0.874
Ramachandran statistics	
* *Favored (%)	96.8
* *Disallowed (%)	0.0
MolProbity clash score	12.82

*Anisotropic diffraction to 2.6 Å in the a and c directions and 3.4 Å in the b direction.

### Molecular Dynamics Simulations

The autoinhibited crystal structure of FLT3 (PDB 1RJB) with and without the juxtamembrane segment present and the co-crystal structure of FLT3 with and without quizartinib present were used as the starting structures in the molecular dynamics simulations. The molecular dynamics calculations were generated using the Gromacs 4.6.2 package [23] with the ff99SB-ILDN force field [24]. The net charge on the proteins was neutralized by adding Na+ and Cl^-^ ions. A box of water molecules was placed on top of the crystallographic coordinates using the TIP3P water model [25]. The systems were relaxed using an initial energy minimization. Then, each protein system was heated to 300 K and was subjected to a 100 ps equilibration at constant number, volume and temperature (NVT). Next, a short 100 ps equilibration was performed at constant number, pressure and temperature (NPT). The final simulations were performed without positional restraints under constant NPT conditions and with v-rescale thermostats in Gromacs 4.6.2. Periodic boundary conditions were imposed on the protein systems. The particle-mesh Ewald summation technique was used to calculate long-range electrostatic interactions [26]. The cut-off for a van der Waals interaction was set at 1 nm. A time step of 2 fs was employed and the structures were stored every 2 ps.

## Results and Discussion

### Quizartinib Binds to an Inactive Conformation of FLT3

**Fig 1:**
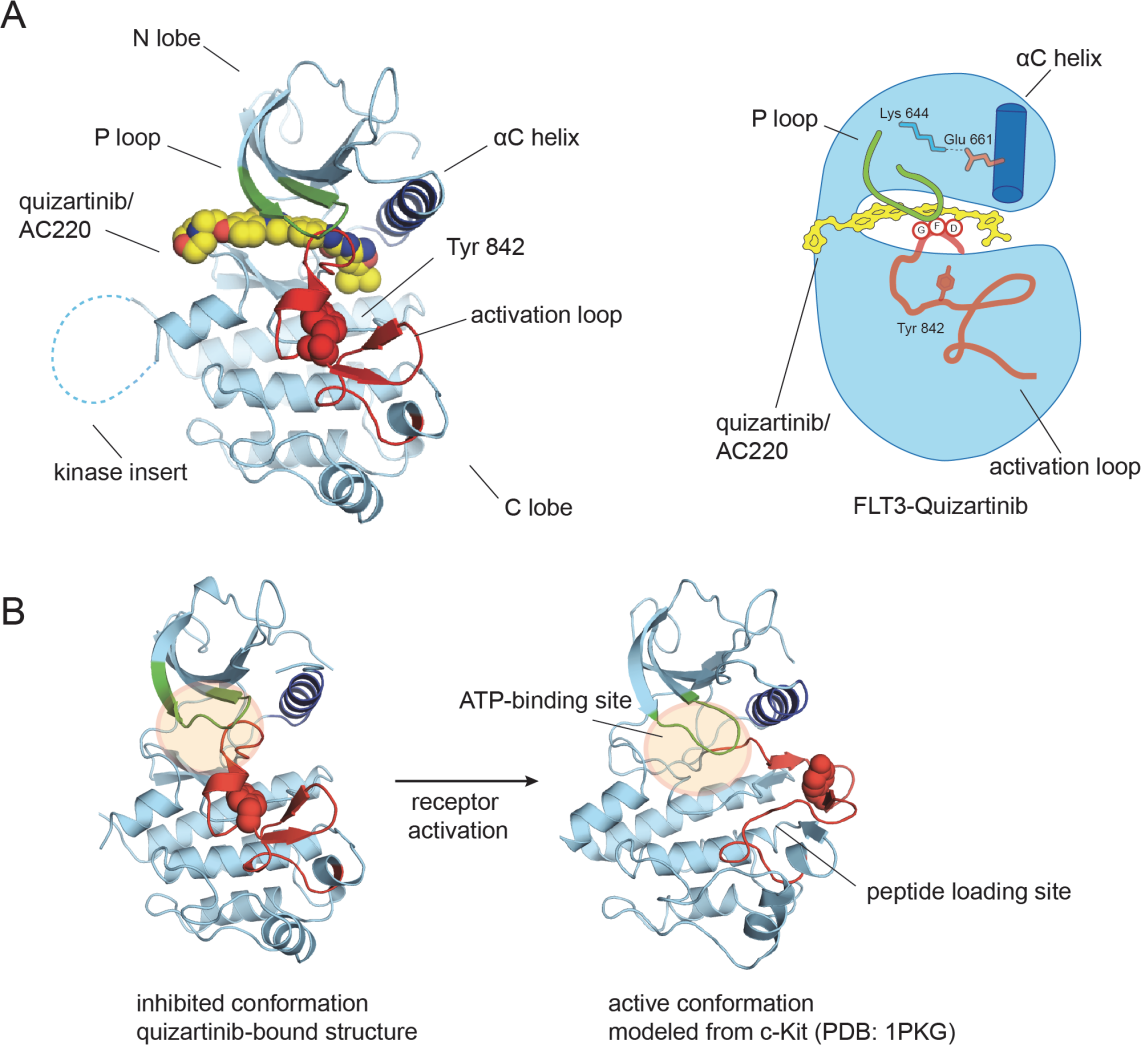
FLT3 with quizartinib bound adopts an inactive conformation. (A) General kinase features are illustrated on the co-crystal structure of FLT3 bound to quizartinib. The kinase is in light blue with the P loop is shown in green, the αC helix is shown in dark blue, and the activation loop is shown in red. (B) Structural changes in kinase domain of FLT3 that occur upon receptor activation are illustrated. The active conformation of FLT3 is modeled from the active c-Kit structure (PDB 1PKG).

As seen in this FLT3 structure, the general kinase fold consists of a smaller N-terminal lobe (N lobe) and an α-helical C-terminal lobe (C lobe) linked by a hinge segment (Fig 1A and 1B). The key conserved structural elements for kinase catalytic activity that lie in between the N lobe and the C lobe include the hinge region, the phosphate-binding loop (P loop), the activation loop, the catalytic loop, and the αC helix [27].

Small molecule inhibitors have trapped many distinct conformational states of kinases. While kinases adopt a common active conformation, there are distinct inactive conformations [28]. In fact, our lab provided a structural basis for the selectivity of the first clinically approved small molecule kinase inhibitor, imatinib, bound to the tyrosine kinase, Abl [29]. Imatinib recognizes a distinct inactive kinase conformation of Abl that is also observed for many receptor tyrosine kinases, including c-Kit and VEGFR. To date, structures of kinases in complex with small molecules have revealed that kinases can adopt active conformations as well as diverse inactive ones [15]. The conformation of the activation loop, including that of a conserved Asp-Phe-Gly element, termed the DFG motif, at the base of the activation loop, and the αC helix in kinases is typically what distinguishes these representative states.

The orientation of the DFG motif is used to classify kinase inhibitors into two representative groups. The aspartic acid residue in the DFG motif, which lies at the N terminus of the activation loop, must be pointed into the active site to coordinate ATP in the active conformation, which is known as the “DFG-in” conformation. Type I inhibitors are classified by this “DFG-in” conformation. Type II inhibitors are defined by the “DFG-out” conformation in which the aspartate is not properly aligned for catalysis [30]. Additional features of a kinase also define the active conformation (Fig 1C). The activation loop must be extended away from the kinase domain to allow ATP and substrate to bind. The αC helix must rotate towards the active site to permit a conserved glutamic acid residue in the αC helix to form a salt bridge to a conserved lysine residue in the active site for ATP coordination.

**Fig 2:**
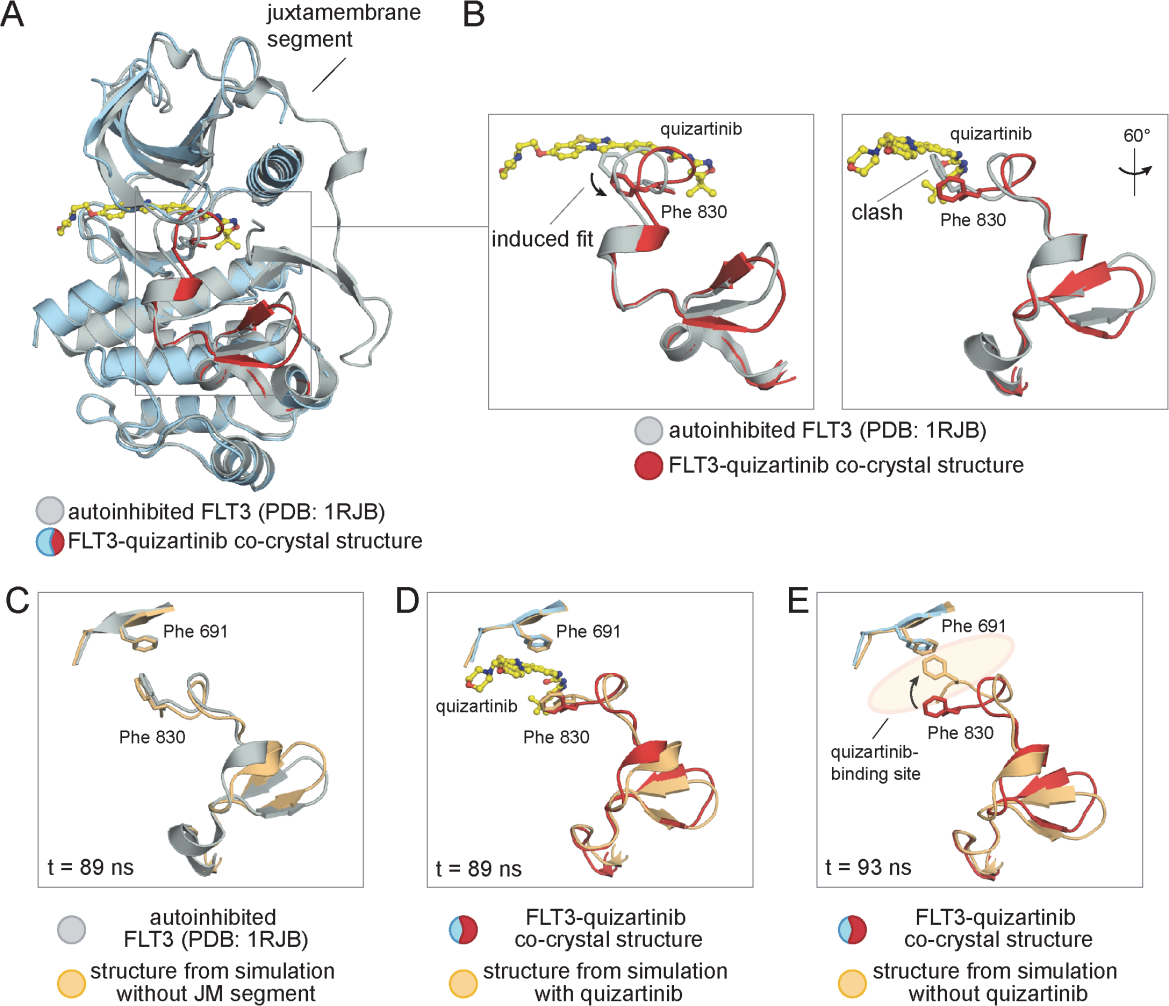
An induced fit of the activation loop of FLT3 with quizartinib bound. (A) An overlay of the kinase domain of FLT3 from the FLT3:quizartinib co-crystal structure (using the color scheme denoted in Fig 1) with the autoinhibited FLT3 (shown in grey, PDB 1RJB). (B) A detailed view of the activation loops on both the FLT3:quizartinib (red) and the autoinhibited FLT3 (grey) structures. The left panel highlights the induced fit of Phe 830 in the DFG motif due to drug binding. Quizartinib (AC220) is included in this zoomed-in view. A 60° rotation of the superposition of the two activation loops is shown in the right panel. (C) A molecular dynamics simulation on the autoinhibited FLT3 with the juxtamembrane segment deleted is illustrated with an overlay of the initial crystal structure in grey and the instantaneous structure at t = 89 ns in light orange. (D) The stability of the activation loop in a molecular dynamics simulation on the FLT3:quizartinib co-complex is illustrated with an overlay of the initial crystal structure in red/ blue and the instantaneous structure at t = 89 ns in light orange. Only the chemical structure of quizartinib (yellow) from the initial structure is shown for clarity. (E) The collapse of the activation loop upon removal of quizartinib in a molecular dynamics simulation (light orange) is illustrated as a comparison to the activation loop in the co-crystal structure (red/ blue).

In our co-crystal structure of quizartinib bound to FLT3, the kinase domain adopts an inactive conformation similar to the autoinhibited structure of FLT3 (PDB 1RJB, Fig 2A) [3]. The two structures can be overlaid very closely, with structural difference restricted to a 4-residue segment of the activation loop (residues 829–832). Excluding the activation loop (residues 829 to 858), the rms deviation in the Cα positions between the two structures is 0.6 Å. While Glu 661 on the αC helix forms a salt bridge with Lys 644 in the active site of FLT3, the activation loop and the DFG motif are not properly oriented to allow substrate phosphorylation to proceed. The activation loop is ordered and folded against the kinase domain to restrict access of substrate. Also, the DFG motif adopts a “DFG-out” orientation, where Phe 830 is pointed into the active site. This general kinase conformation resembles that of the inactive forms of related receptor tyrosine kinases, such as c-Kit and VEGFR, and falls into a general class termed the “Abl-like” inactive conformation [4,7,29].

The structural differences between the activation loops in autoinhibited FLT3 and the quizartinib:FLT3 co-complex appears to be induced by drug binding (Fig 2B). Even upon deleting the juxtamembrane segment, the conformation of the activation loop of the autoinhibited FLT3 remains stable in molecular dynamics simulations over 100 ns (Fig 2C). While the activation loop remains folded against the kinase domain in this simulation, the αC helix does swing slightly in towards the active site. Quizartinib stabilizes a similar inactive conformation of FLT3 in molecular dynamics simulations (Fig 2D). In this case, the activation loop, the αC helix, and the DFG motif remain stable throughout the course of the simulation. However, if quizartinib is removed from the co-crystal structure, the activation loop collapses into the active site during simulations, and Phe 830 in the DFG motif forms hydrophobic interactions with the gatekeeper residue Phe 691 (Fig 2E). The gatekeeper residue is so termed because it is a key feature of small molecule specificity determination in the kinase active site [31]. The simulations indicate that FLT3 adopts a meta-stable state in the co-crystal structure that is stabilized by quizartinib. Both the conformation of FLT3 in the co-crystal structure and the collapsed conformation adopted in the simulation after quizartinib is deleted are distinct from the conformation of the autoinhibited FLT3 (PDB 1RJB).

This FLT3 conformation with the DFG motif in the “DFG-out” orientation would characterize quizartinib as a type II inhibitor. This inhibitor class is believed to have greater selectivity relative to type I inhibitors since kinases have similar active conformations but distinct inactive conformations [32]. Consistent with our structural observations, quizartinib exhibits a high degree of potency and selectivity for FLT3 [17,18].

### Interactions between quizartinib and FLT3

**Fig 3:**
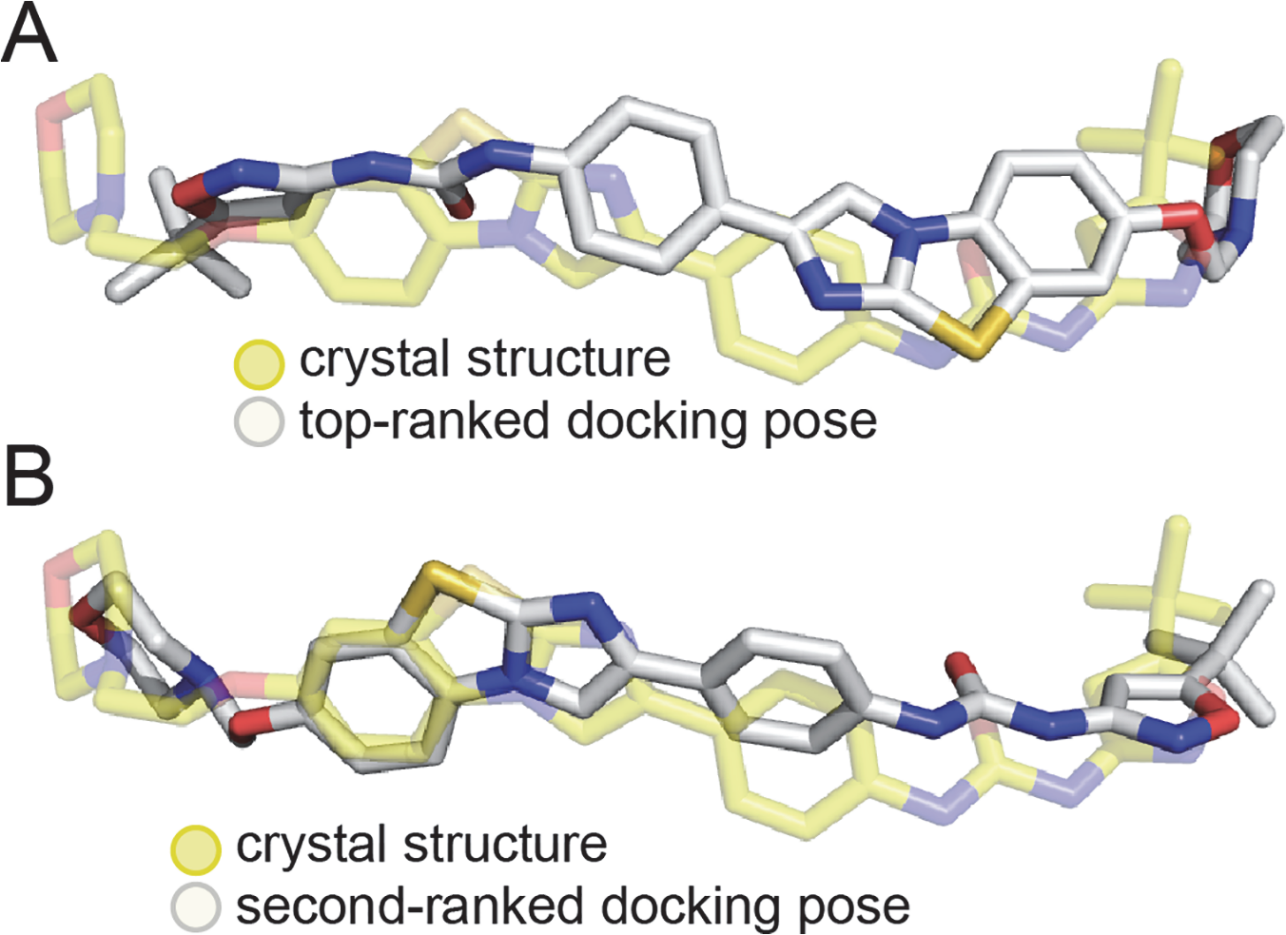
Quizartinib in the FLT3 co-complex matches the second-ranked docking pose. (A) An overlay of quizartinib from the co-crystal structure (yellow) with the top-ranked docking pose (white) is shown. (B) An overlay of quizartinib from the co-crystal structure (yellow) with the second-ranked docking pose (white) is shown.

**Fig 4:**
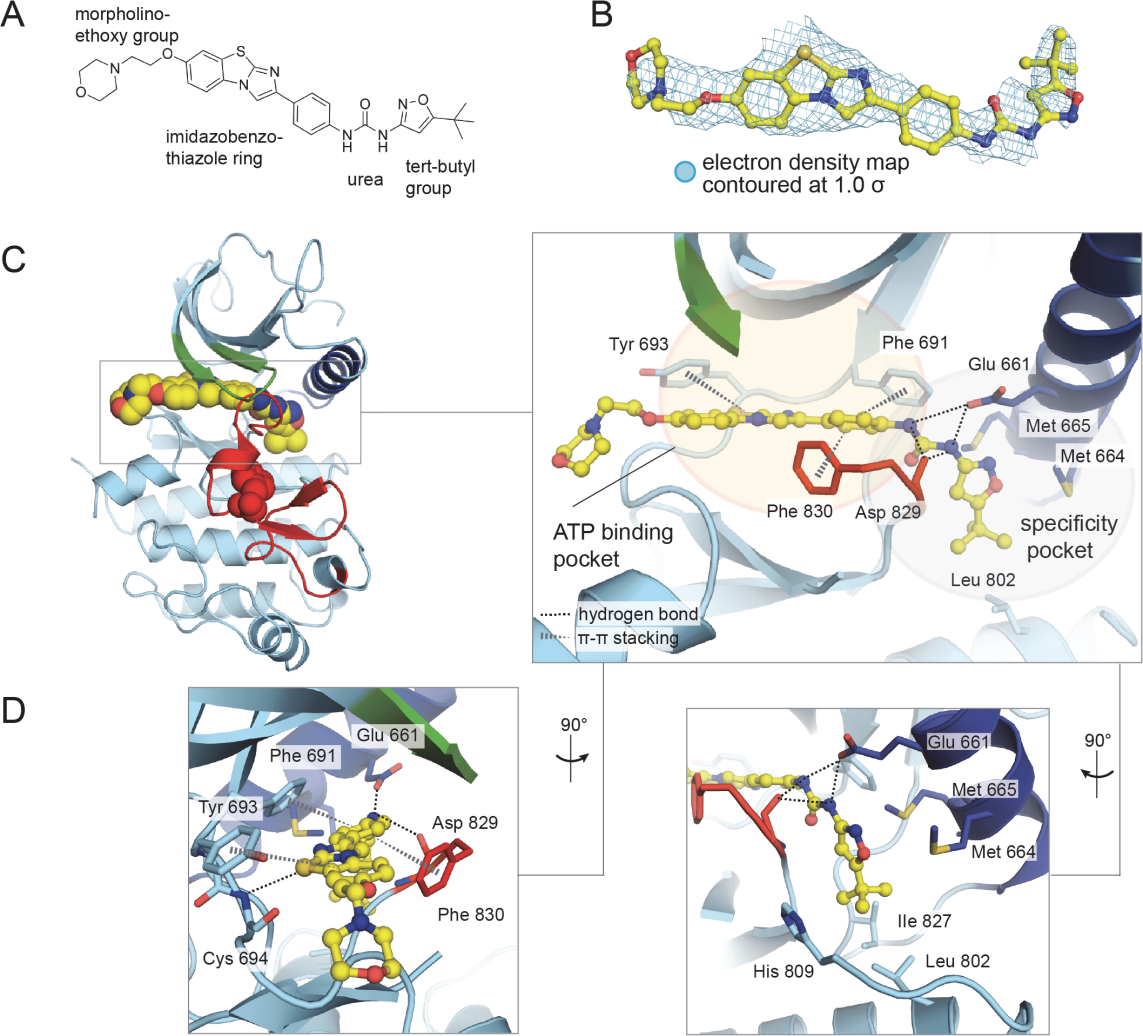
Interactions between FLT3 and quizartinib. (A) Chemical structure of quizartinib (AC220). (B) An unbiased electron density map (2mF_O_-DF_C_) of quizartinib (yellow) contoured at 1.0 σ (light blue). A simulated annealing refinement in Phenix on FLT3 with quizartinib deleted resulted in a model that was used to calculate the electron density for quizartinib. The simulated annealing was performed with an initial temperature of 5000 K to a final temperature of 300 K over 50 steps. A superposition of quizartinib with this unbiased electron density map for the compound is shown for clarity. (C) The structure of the FLT3 kinase domain bound to quizartinib (left) with a zoomed-in view of the active site (right). (D) A detailed view of the interactions between FLT3 and quizartinib.

Our previous report utilizing molecular docking suggest that the top-ranked binding pose of quizartinib in the active site of FLT3 is rotated 180° relative to the structure determined here (Fig 3A) [19]. However, the second-ranked pose in the docking calculation is consistent with the quizartinib-FLT3 co-crystal structure (Fig 3B). An unbiased electron density map of the compound in the active site of FLT3, which was generated by performing a simulated annealing refinement on the FLT3 structure with the compound deleted, clearly reveals the orientation of quizartinib (Fig 4A and 4B).

One favorable interaction observed in the quizartinib-FLT3 co-crystal structure, in contrast to the top-ranked docking pose, is between the tert-butyl substituent on the isoxazole ring of quizartinib and a hydrophobic pocket on FLT3 (Fig 4C). The pocket includes Met 664 and Met 665 on the αC helix, Ile 674 in the N lobe, Leu 802 in the C lobe, and Ile 827, which is immediately N terminal to the DFG motif (Fig 4D). Interestingly, if the co-crystal structure of VEGFR bound to sorafenib is superimposed on the co-crystal structure of FLT3 bound to quizartinib, the tert-butyl substituent on quizartinib sits in a similar pocket on FLT3 in comparison to the hydrophobic trifluoromethyl group on sorafenib in the active site of VEGFR [4]. In comparison, the top ranked docking result placed a hydrophilic morpholinoethoxy group into this pocket on FLT3. Previous structure-activity relationship studies also indicate that the morpholinoethoxy group does not affect the potency of quizartinib, but rather serves to improve its aqueous solubility [18]. This is consistent with this group pointing out of the active site into solvent, as seen in our crystal structure.

Diaryl ureas were identified as scaffolds that elicited potent inhibition of FLT3 [16]. The co-complex of quizartinib with FLT3 reveals important interactions that this moiety makes in the kinase active site. The carbonyl group of the urea forms hydrogen bond interactions with the backbone nitrogen of Cys 828. Further, the nitrogens in the urea moiety form hydrogen bond interactions with Glu 661 in the αC helix (Fig 4D).

The central para-substituted phenyl ring on quizartinib makes key interactions in the kinase active site of FLT3. This ring is sandwiched between the gatekeeper residue, Phe 691, and the phenylalanine residue in the DFG motif, Phe 830. These two residues both form π-π interactions with this ring in quizartinib, which presumably contributes to the stability of quizartinib in the active site.

The imidazobenzothiazole ring on quizartinib was previously predicted from molecular docking to form parallel π-π stacking interactions with the gatekeeper residue, Phe 691; however, the phenyl ring of quizartinib occupies this position in the co-crystal structure, while the imidazobenzothiazole ring makes interactions with the hinge region. The sulfur atom in the imidazobenzothiazole ring forms a weak hydrogen bond with the backbone nitrogen of Cys 694. Additional weak π-π stacking interactions between Tyr 693 in the hinge region and the imidazobenzothiazole ring are also observed in the co-crystal structure.

### The juxtamembrane segment of FLT3 is not compatible with quizartinib binding

**Fig 5:**
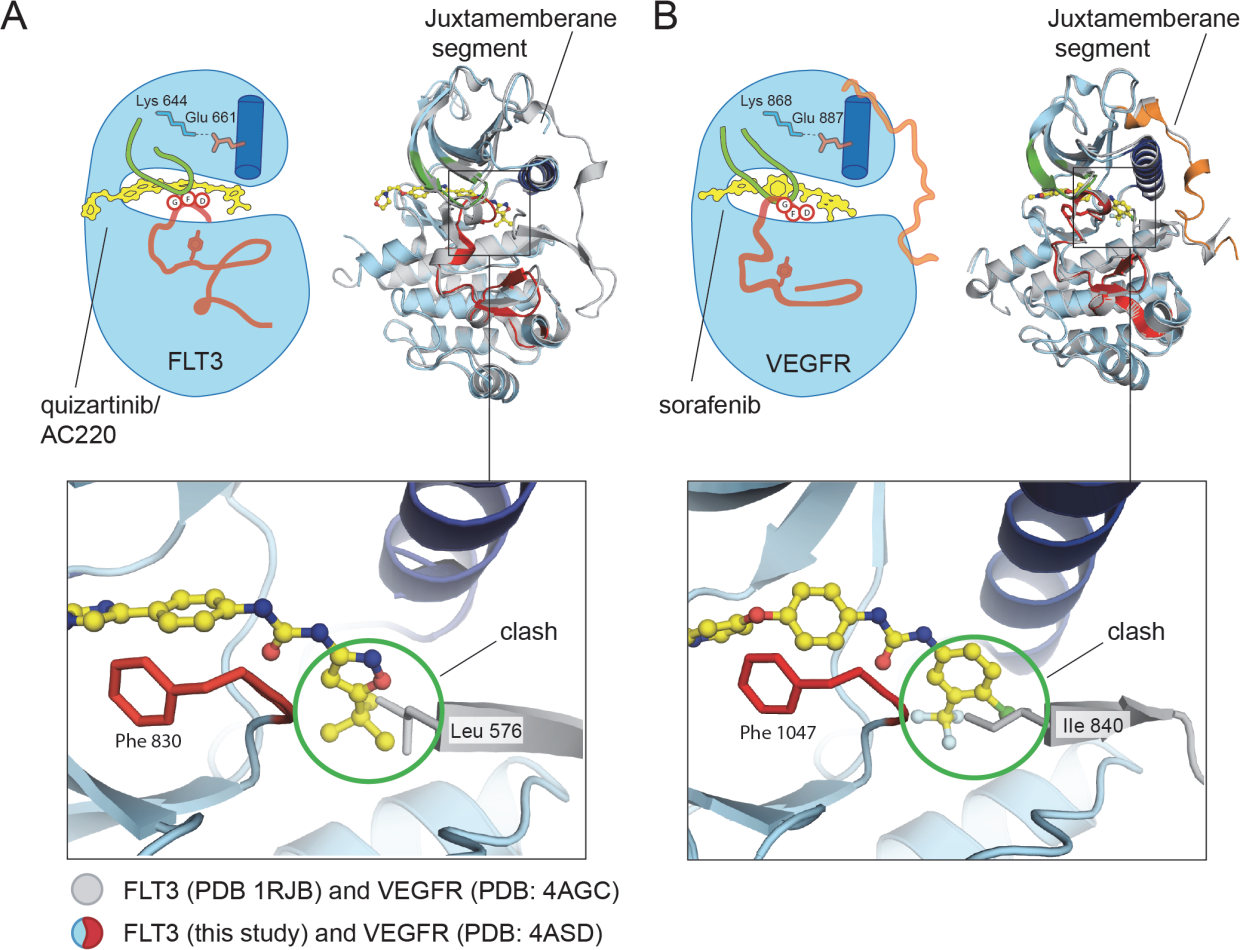
The juxtamembrane segment of FLT3 clashes with quizartinib. (A) An overlay of the FLT3:quizartinib crystal structure (color scheme depicted in Fig 1A) with the autoinhibited FLT3 (shown in grey, PDB 1RJB). The zoomed-in view of the juxtamembrane segment highlights clashes between the juxtamembrane segment and quizartinib. (B) An overlay of the crystal structure of the VEGFR2 kinase domain bound to sorafenib (shown in red/ blue, PDB 4ASD) and the VEGFR2 kinase domain bound to axitinib (shown in grey, PDB 4AGC). Only sorafenib is illustrated in the active site for clarity. The juxtamembrane segment from the VEGFR2:sorafenib co-crystal structure is shown in orange and is extended away from the active site. The juxtamembrane segment from the VEGFR:axitinib co-crystal structure remains bound to the kinase domain. The detailed view illustrates similar clashes between sorafenib and the VEGFR2 bound conformation of the juxtamembrane segment.

While the autoinhibited FLT3 (PDB 1RJB) and the co-crystal structure of FLT3 with quizartinib both adopt an inactive “Abl-like” structure, one distinct feature is the absence of the juxtamembrane segment in the co-crystal structure. This segment was included in the crystallization construct, but no interpretable electron density is visible for it. A superposition of the juxtamembrane segment from the autoinhibited structure onto the quizartinib-FLT3 co-complex illustrates that quizartinib binding is not compatible with the juxtamembrane segment being folded onto the kinase domain (Fig 5A). This superposition indicates that Leu 576 on the juxtamembrane segment would clash with quizartinib. Notably, the conformation of the juxtamembrane segment in the autoinhibited FLT3 (PDB 1RJB) structure would not be compatible with the crystal packing in the FLT3:quizartinib structure, which may also contribute to the lack of electron density for this region.

Similar clashes between small molecule inhibitors and the autoinhibitory juxtamembrane segment have been reported for related receptor tyrosine kinases, including c-Kit and VEGFR. For example, the clinically-approved inhibitor imatinib is not compatible with binding of the juxtamembrane segment in c-Kit, and sorafenib is not compatible with this segment in VEGFR (Fig 5B) [4,7]. While these clashes have been observed for receptor tyrosine kinases, small molecule inhibitors do not always disrupt interactions between the kinase domain and the juxtamembrane segment. For example, another VEGFR inhibitor, axitinib, makes favorable interactions with the juxtamembrane segment in the kinase active site [4].

### Resistance mutations restrict quizartinib binding to FLT3

**Fig 6:**
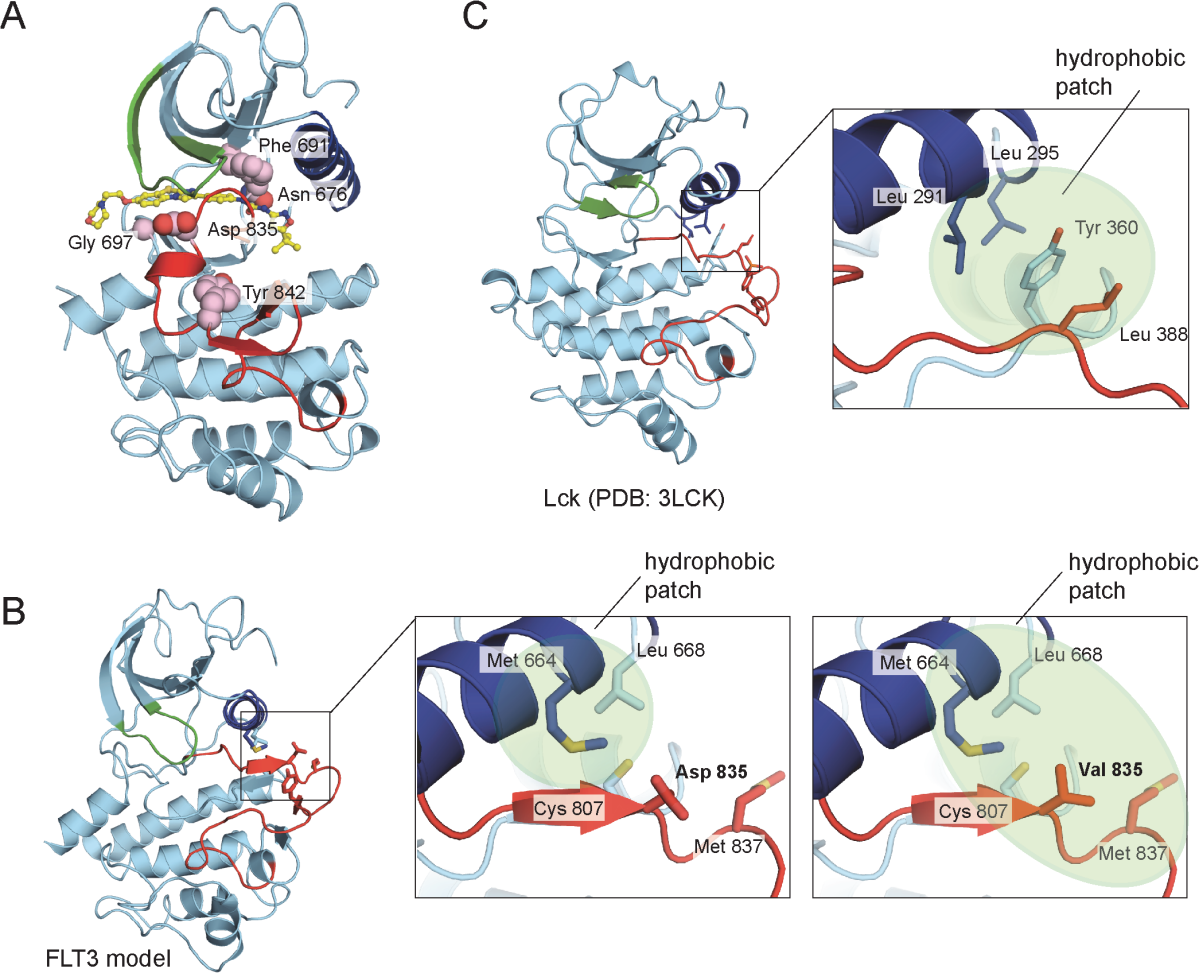
Mutations in the kinase domain of FLT3 in acute myeloid leukemia (AML). (A) Mutations observed in AML (pink spheres) that show resistance to quizartinib are mapped onto the kinase domain of FLT3. (B) The left panel illustrates the position of a hydrophobic pocket on a homology model of FLT3, which was generated from the active conformation of c-Kit (PDB 1PKG). The zoomed-in view in the middle panel illustrates the detailed interactions between the activation loop and a hydrophobic pocket on the model of FLT3. The right panel highlights an extension of the hydrophobic patch with the disease mutation, D835V. (C) A similar interaction between a hydrophobic pocket on the kinase domain and the activation loop is observed in the crystal structure of the active conformation of LCK (PDB 3LCK).

Activating mutations in FLT3 are present in a significant population of AML patients, and are associated with lower response rates to traditional treatment regimens. Quizartinib is a promising therapy for these patients, but additional resistance mutations arise. One of the most common of these resistance mutations occurs at the gatekeeper residue, Phe 691, which is mutated to a leucine or isoleucine residue in patients that relapsed [33]. This mutation would disrupt a key interaction between the gatekeeper phenylalanine and a phenyl ring on quizartinib (Fig 6A).

Importantly, mutation of Asp 835 is not only observed as a resistance mutation to quizartinib treatment, but also as an activating mutation in patients diagnosed with AML. This residue is often mutated to hydrophobic residues, including valine, phenylalanine, and tyrosine. Asp 835 in the co-crystal structure of quizartinib bound to FLT3 does not make direct interactions with the drug. This mutation is located in the activation loop of FLT3, and may stabilize the activation loop in an active conformation, extended away from the kinase domain. Mutations in the activation loop of a kinase can alter the balance between the active and inactive state [34].

While an active conformation of FLT3 is not yet reported, an active conformation of a related family member, c-Kit, which has 65% sequence identity with the kinase domain of FLT3, has been reported (PDB 1PKG) [35]. We constructed a homology model of FLT3 using the c-Kit structure as a template [36]. Interestingly, Asp 835 in the model for the active conformation lies adjacent to a hydrophobic patch on FLT3 (Fig 6B). Mutation of Asp 835 to a more hydrophobic residue could promote interactions with this pocket and stabilize the activation loop in an extended conformation. In a structure of the tyrosine kinase, Lck, where the kinase domain adopts an active conformation, a leucine residue occupies this position in the activation loop and interacts with a similar hydrophobic pocket (Fig 6C) (PDB 3LCK). Mutation of this leucine residue in Lck to an aspartic acid residue results in a less active Lck variant, which is consistent with observations for FLT3 [37]. Stabilizing the active conformation of FLT3 in this fashion would disfavor quizartinib binding since the drug recognizes an inactive conformation.

## Conclusions

Since receptor tyrosine kinases occupy a central role in the initiation of cellular signaling cascades, their activity often becomes deregulated in cancer [14,38]. Thus, many drug discovery programs have focused on the development of inhibitors targeting this family. In particular, FLT3 has been implicated as a driver mutation in AML [10]. Quizartinib is a second generation FLT3 inhibitor that has shown promising results in the clinic against AML.

Here, we determined the co-crystal structure of FLT3 bound to quizartinib. FLT3 adopts an “Abl-like” inactive conformation with quizartinib bound. The DFG motif adopts a “DFG-out” conformation, the activation loop is folded back onto the kinase domain to mimic peptide substrate binding, and Glu 661 on the αC helix forms a salt bridge with Lys 644 in the active site. A previous structure of autoinhibited FLT3 shows that the juxtamembrane segment folds back onto the kinase domain and stabilizes the kinase in a similar inactive state [3]. The juxtamembrane segment, however, is not observed in our structure, and would not be compatible with quizartinib in the active site of FLT3.

A rearrangement of the juxtamembrane segment in the autoinhibited FLT3 would be required for quizartinib to bind, similar to the rearrangement described for the related receptor tyrosine kinase, VEGFR, for sorafenib to bind [4]. In fact, quizartinib is more potent against FLT3 variants that contain the ITD activating mutations in the juxtamembrane segment, which are believed to release the inhibitory interactions that the juxtamembrane makes. Although the ITD mutation is one of the most common mutations observed in AML, restricting wild-type FLT3 activation would also be important to curb disease progression. Small molecule inhibitors that are compatible with the juxtamembrane segment conformation in the autoinhibited VEGFR were found to be more potent inhibitors relative to sorafenib [39]. Similar strategies may yield more potent and selective inhibitors against wild-type, autoinhibited FLT3.

While quizartinib is a promising treatment for AML, resistance mutations in FLT3 have been identified in response to this drug [19]. Mutation of the gatekeeper residue, F691L, and mutations in the activation loop lead to resistance. It is hoped that our structure of FLT3 with quizartinib bound will aid efforts to design new inhibitors that lead to useful therapies to treat AML patients that develop resistance to quizartinib.
